# Modulation of osteoblast differentiation and function by the P2X4 receptor

**DOI:** 10.1007/s11302-022-09887-x

**Published:** 2022-08-17

**Authors:** Isabel R. Orriss, Bethan K. Davies, Lucie E. Bourne, Timothy R. Arnett

**Affiliations:** 1 Department of Comparative Biomedical Sciences, Royal Veterinary College, Royal College Street, London, NW1 0TU UK; 2 Department of Cell and Developmental Biology, University College London, London, WC1E 6BT UK

**Keywords:** P2X4 receptor, Osteoblast, Differentiation, Bone formation

## Abstract

Bone cells are known to express multiple P2 receptor subtypes, and the functional effects of receptor activation have been described for many of these. One exception is the P2X4 receptor, which despite strong expression in osteoblasts and osteoclasts, has no defined functional activity. This study used the selective P2X4 receptor antagonists, 5-BDBD and PSB-12062, to investigate the role of this receptor in bone. Both antagonists (≥ 0.1 μM) dose-dependently decreased bone formation by 60–100%. This was accompanied by a ≤ 70% decrease in alkaline phosphatase activity, a ≤ 40% reduction in cell number, and a ≤ 80% increase in the number of adipocytes present in the culture. The analysis of gene expression showed that levels of osteoblast marker genes (e.g. *Alpl, Bglap*) were decreased in 5-BDBD treated cells. Conversely, expression of the adipogenic transcription factor *PPARG* was increased 10-fold. In osteoclasts, high doses of both antagonists were associated with a reduction in osteoclast formation and resorptive activity by ≤ 95% and ≤ 90%, respectively. Taken together, these data suggest that the P2X4 receptor plays a role in modulating bone cell function. In particular, it appears to influence osteoblast differentiation favouring the osteogenic lineage over the adipogenic lineage.

## Introduction

The role of purinergic signalling and extracellular nucleotides in the regulation of bone cell function has been studied extensively over the last 20 years [1–3]. Osteoblasts, osteoclasts, and osteocytes express multiple P2 receptor subtypes and functional studies have shown that extracellular nucleotides including ATP, UTP, and ADP can influence bone cell differentiation, gene expression, survival and function [1–3]. Furthermore, several P2 receptor knockout mouse models display skeletal changes [4–7].

The P2 receptors are split into the ionotropic P2X receptors and the metabotropic P2Y receptors. P2X receptors are ligand-gated ion channels, which are activated by ATP (P2X1-7), whilst the P2Y receptors are G-protein linked and are activated by a range of extracellular nucleotides including ATP, ADP, and UTP [8]. Bone cells express the majority of P2X and P2Y receptor subtypes, but only some of these receptors have been associated with specific functional effects in bone. For example, the P2Y_2_ receptor has been associated with regulating ATP release and bone mineralisation [6, 9, 10]. Whereas the P2Y_1_, P2Y_6_, and P2Y_12_ receptors are thought to regulate osteoclast formation and activity [7, 11, 12]. In terms of the P2X receptors, the P2X7 receptor remains the most investigated; however, studies report conflicting effects on bone cell function [13–18].

The P2X4 receptor displays widespread tissue expression and can mediate a range of functional effects including neuropathic pain, inflammation, and responses to alcohol [19]. To date, there is little information regarding the effects of P2X4 receptor stimulation on bone cell function. Previous work has shown that both osteoblasts and osteoclasts express the P2X4 receptor [12, 15, 20–23]. In osteoblasts, the comparative analysis showed that this receptor was the most strongly expressed P2 receptor at both mRNA and protein levels. Furthermore, expression of the P2X4 receptor was influenced by cellular differentiation with the highest levels seen in mature osteoblasts [15]. Early work in osteoclasts attributed an ATP-induced cation influx and depolarisation to the P2X4 receptor [22]. However, a subsequent study suggested that the nucleotide-induced increase in intracellular calcium arose primarily from P2Y receptor activation [24]. In osteoblast-like cells, the P2X4 receptor has been suggested to be involved in ATP-induced proliferation [25]. Interestingly, a study by Wesselius et al. [26] demonstrated an association between polymorphisms in the P2X4 receptor and the risk of osteoporosis, with the Tyr315Cys polymorphism showing an increased risk of osteoporosis. The P2X4 receptor has also been associated with the regulation of chondrogenesis [27]. The P2X4 receptor knockout mouse model was first developed in 2006 and has been used in a wide range of studies [28]. A recent report found that P2X4 receptor knockout mice displayed increased cortical and trabecular bone mineral density [29].

The P2X4 receptor is activated by the universal P2 receptor agonist, ATP, and also the nonselective analogue α,β-meATP. In recent years, several selective antagonists for the P2X4 receptor have been developed including 5-BDBD and PSB-12062. The aim of this investigation was to use these antagonists to probe the role of the P2X4 receptor in osteoblast and osteoclast function.

## Methods

### Reagents

All tissue culture reagents were purchased from Thermo Fisher Scientific UK; unless mentioned, all chemicals were obtained from Sigma-Aldrich (Poole, UK).

### Osteoblast bone formation assay

Primary osteoblasts were isolated from 2- to 3-day-old Sprague–Dawley rats by trypsin collagenase digestion as previously described [30, 31]. The basal medium for cell culture was Dulbecco’s Modified Essential Medium supplemented with 10% foetal calf serum (FCS), 2 mM L-glutamine, 100 U/ml penicillin, 100 μg/ml streptomycin, and 0.25 μg/ml amphotericin (complete mixture abbreviated to DMEM). Cells were cultured for up to 14 days in DMEM supplemented with 2 mM β-glycerophosphate, 50 μg/ml ascorbate and 10 nM dexamethasone, with half medium changes every 3 days. Time points in osteoblast cultures were defined as proliferating (day 4), differentiating (d7), and mature, bone-forming (day 14). All experiments were performed on cells that were isolated, expanded, and plated: the cells were not passaged at any stage. Osteoblasts were cultured with 0.1–10 μM 5-BDBD, 0.1–5 μM PSB-12062 or 1–100 μM nucleotides (ATP, UTP, ADP, α,β-meATP) for the duration of the culture. Since the antagonists were dissolved in DMSO, all control wells contained a DMSO vehicle control.

To assess bone formation, experiments were terminated by fixing the cells in 2.5% glutaraldehyde for 5 min. Cell culture plates were imaged at 800 dpi using a flatbed scanner (Epson Perfection Photo) and the total area of bone nodules formed was quantified by image analysis, as previously described [30, 31].

### Osteoclast formation assay

Osteoclast precursor cells were isolated from the long bones of 6- to 8-week-old C57Bl/6 mice as described previously [32]. Basal cell culture medium was a Minimum Essential medium supplemented with 10% FCS, 2 mM L-glutamine, 100 U/ml penicillin, 100 μg/ml streptomycin, and 0.25 μg/ml amphotericin (complete mixture abbreviated to MEM). In a 96-well tray, cells were plated onto 5-mm dentine discs in MEM supplemented with 100 nM PGE_2_, 200 ng/ml M-CSF, and 3 ng/ml RANKL (R&D Systems Ltd, Abingdon, UK). After 24 h, discs containing adherent osteoclast precursors were transferred to 6-well trays (4 discs/well in 4 ml medium) for a further 6 days. The culture medium was acidified to pH 7.0 by the addition of 10 meq/l H^+^ (as HCL) on day 7 to activate osteoclasts to resorb [32, 33]. 5-BDBD (0.1–10 μM) or PSB-12062 (0.1–5 μM) was added for the duration of the culture.

Dentine discs were fixed in 2.5% glutaraldehyde and stained to demonstrate tartrate-resistant acid phosphatase (TRAP). Osteoclasts were defined as TRAP-positive cells with 2 or more nuclei and/or clear evidence of resorption. Osteoclast number and resorption pit formation were assessed “blind” using transmitted and reflective light microscopy, respectively, as previously described [32].

### Total RNA extraction and DNase treatment

Osteoblasts were cultured for 7 or 14 days and osteoclasts for 5, 7, or 9 days before total RNA was extracted using Qiazol® reagent (Qiagen Ltd, Manchester, UK) according to the manufacturer’s instruction. Osteoblasts were also cultured for 7 or 14 days in the presence of 10 μM 5-BDBD before RNA collection. Extracted RNA was treated with RNase-free DNase I (35 U/ml) for 30 min at 37 °C. The reaction was terminated by heat inactivation at 65 °C for 10 min. Total RNA was quantified spectrophotometrically by measuring absorbance at 260 nm (Nanodrop 1, Thermo Fisher UK). RNA was stored at − 80 °C until amplification by qRT-PCR.

### Quantitative real-time polymerase chain reaction (qRT-PCR)

Osteoblast and osteoclast RNA (50 ng) was transcribed and amplified using the qPCRBIO SyGreen one-step qRT-PCR kit (PCR Biosystems, London, UK), which allows cDNA synthesis and PCR amplification to be carried out sequentially. qRT-PCR was performed according to the manufacturer’s instructions with initial cDNA synthesis (45 °C for 10 min) and reverse transcriptase inactivation (95 °C for 2 min) followed by 40 cycles of denaturation (95 °C for 5 s) and detection (60 °C for 30 s). All reactions were carried out in triplicate using RNA derived from 4 to 5 different cultures. Data were normalised to β-actin and analysed using the ΔΔCt method [34]. Expression of the P2X and P2Y receptors as well as osteoblast (tissue nonspecific alkaline phosphatase (*Alpl),* type 1 collagen (*Col1α1*), osteocalcin (Ocn, *Bglap),* ecto-nucleotide pyrophosphatase/phosphodiesterase 1 (NPP1, *Enpp1*)), and adipocyte (PPARγ (*PPARG*)) marker genes was investigated. Primer sequences are shown in Table 1.

**Table 1:** List of primer sequences

Gene	Primer sequence (5′-3′)	
*βactin*	S	gcc ttc ctt cct ggg tat gg
	AS	gag gtc ttt acg gat gtc aac g
*P2XR1*	S	cgg act gta tgg gga gaa ga
	AS	tcc caa ac acct tga aga gg
*P2XR2*	S	att cag tct cat tcc cac ca
	AS	atc cag tca cac agg aag ga
*P2XR3*	S	tct tga ggg tag ggg atg tg
	AS	cac acc cag ccg atc tta at
*P2XR4*	S	atc cct tct gcc cca tat tc
	AS	ttg cag tcc cac ttg atc tg
*P2XR5*	S	ttg aat ggg act gtg acc tt
	AS	ttg tac cca gag gag atg ga
*P2XR6*	S	cgg ttt cta ctg gag gac ca
	AS	agc agg gtt agc agg tga ga
*P2XR7*	S	cag ggg gaa gta gtc aac ct
	AS	cag ttc gtc tcc tgc agt tt
*P2YR1*	S	tgc cat tta tgt cag tgt gc
	AS	tcc cag tgc cag agt aga ag
*P2YR2*	S	cct gga ata agt acc atc aat gg
	AS	agc agc aca tac ttg aag tc
*P2YR4*	S	gtc ttt gct gtc tgc ttc gt
	AS	atg aca gtc agc ttg caa ca
*P2YR6*	S	tcc tca cct gca tta gct tc
	AS	gac tcc aca tac cac cca ag
*P2YR13*	S	aag aag ttc acc cgg aag gt
	AS	ctg act gct gtg gtg ctc at
*PPARG*	S	tgc cta tga gca ctt cac ac
	AS	atc cat cac aga gag gtc ca
*Bglap*	S	agc tca acc cca aat gtg ac
	AS	act ctc cag gac tcg acc ct
*Alpl*	S	aaa cct aga cac aag cac tc
	AS	tcc gat tca act cat act gc
*Col1α1*	S	ggg aca cag agg ttt cag tgg
	AS	agc tcc att ttc acc agg act g
*Enpp1*	S	aga cca cac ttt tac act ctg
	AS	gat gac ctc act gct tac tg
*βactin *(mouse)	S	gat ctg cga cca cac ctt ct
	AS	ggg gtg ttg aag gtc tca aa
*P2XR4*(mouse)	S	gca ccc tcc acc atc tct aa
	AS	aa acct ctt gcc aga agc aa

### Immunofluorescence

Osteoclasts and osteoblasts were cultured on sterile 1 cm discs, cut from Melinex (Du Pont, Dumfries, UK), for 7 or 4–14 days, respectively. Cells were fixed with 4% paraformaldehyde in 0.1 M phosphate buffer for 20 min at room temperature, washed 3 × 5 min in PBS and stored at 4 °C in PBS until staining. Each disc was incubated with a 2% BSA in PBS blocking solution for 1 h. Discs were then incubated overnight at 4 °C in the primary antibody solution (P2X4, 1:200 in 2% BSA in PBS). Cells were washed 3 × 5 min in PBS before incubation for 1 h with a Cy3-labelled secondary antibody solution (1:400 in PBS with 1% BSA). After three further 5-min PBS washes, discs were mounted onto microscope slides using Prolong™ Diamond Antifade Mountant with DAPI (Thermo Fisher Scientific, UK) and viewed by fluorescence microscopy (Cy3 absorbance and emission at 550 nm and 570 nm, respectively).

### Alkaline phosphatase (TNAP) activity 

Osteoblasts were cultured for up to 7 or 14 days in a medium supplemented with 0.1–1 μM 5-BDBD/PSB-12062. TNAP activity was measured in cell lysates using a colorimetric assay (Anaspec, CA, USA), as previously described [30]. TNAP activity was normalised to cell protein using the Bradford assay.   

### Cell number and viability assay

Osteoblasts were cultured for 4, 7, or 14 days in a medium supplemented with 0.1–1 μM 5-BDBD/PSB-12062. Cell number and viability was determined using the CytoTox 96**®** colourimetric cytotoxicity assay (Promega UK, Southampton UK), as described previously [35]. Cell supernatants were collected to determine medium LDH levels (cell viability). To establish total cellular LDH levels (cell number), cells were lysed with 1% Triton X-100 in water (lysis buffer, 15 μl/ml of medium) for 1 h. The LDH content of the supernatants and cell lysates were measured colourimetrically (495 nm) as per the manufacturer’s instructions. A standard curve for determining cell number was constructed using cells seeded at 10^2^ to 10^6^/well. Cell viability (given as a percentage of dead cells) was calculated by expressing medium LDH as a percentage of the total cellular LDH.

### Oil red O staining for adipocytes

This assay was based on the method originally described by Ramirez-Zacarias [36]. Osteoblasts were cultured with 1–100 μM ATP/ADP/UTP or 0.1–10 μM 5-BDBD/PSB-12061 for 14 days. Cells were fixed with 2.5% glutaraldehyde for 5 min, washed with 60% isopropanol, and allowed to air dry. The oil red O stock solution (0.35% w/v in isopropanol) was diluted to a working solution (6 parts stock: 4 parts dH_2_O) and added to the fixed cells for 10 min. Following four washes with distilled water, cell layers were left to dry before imaging. The amount of oil red O staining was quantified by eluting the stain with 100% isopropanol (500 μl/well for 10 min) and reading the optical density at 500 nm.

### Statistics

Data were analysed using GraphPad Prism 8 software (San Diego, CA). In vitro results represent data from 4 to 6 individual experiments; each experiment was performed using cells isolated from different animals. Within each experiment, each group contained 3–6 technical replicates. To account for the inherent variation of using primary cells isolated from different animals, all in vitro data were analysed using a randomised block ANOVA, followed by Fisher’s LSD post hoc test as described by Festing [37].

## Results

### Bone cells express the P2X4 receptor

**Fig. 1:**
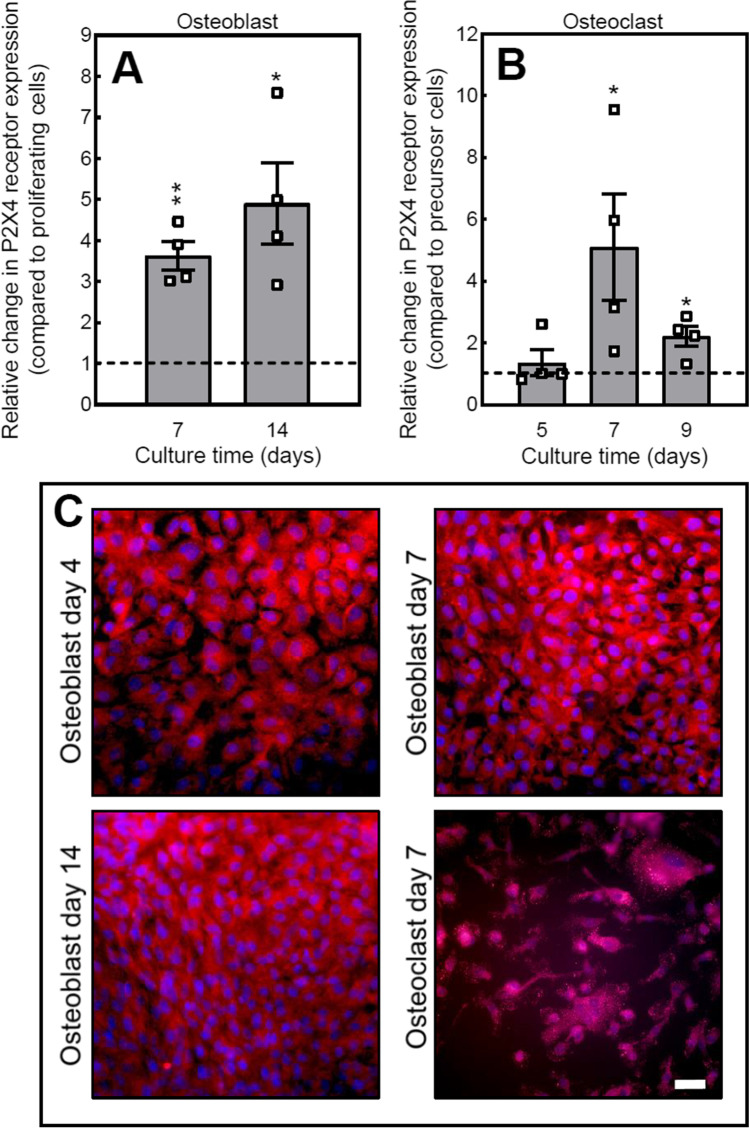
Expression of the P2X4 receptor by bone cells. Analysis of gene expression shows (**A**) increased expression of the P2X4 receptor in differentiating (day 7) and mature, mineralising (day 14) osteoblasts, relative to proliferating cells (day 4; dotted line). (**B**) Osteoclast expression of the P2X4 receptor is increased in mature cells, relative to precursor cells (day 3; dotted line). Data shown as mean ± SEM (*n* = 4 RNA sets), * = *p* < 0.05, ** = *p* < 0.01. (**C**) Immunofluorescence images showing widespread protein expression of the P2X4 receptor in osteoblasts and osteoclasts. Scale bar = 50 μm

The qPCR analysis showed the expression of P2X4 receptor mRNA at all stages of osteoblast differentiation; levels were ≤ 4-fold and ≤ 5-fold in differentiating and mature, mineralising osteoblast, respectively, compared to proliferating osteoblasts (Fig. 1A). P2X4 mRNA expression was also present at all stages of osteoclast differentiation with the highest levels observed in mature cells (Fig. 1B). Immunofluorescent staining showed that P2X4 receptor protein was expressed in osteoblasts at all stages of the culture and in mature osteoclasts (Fig. 1C).

### The P2X4 receptor antagonists, 5-BDBD and PSB-12062, inhibit bone formation and reduce TNAP activity

**Fig. 2:**
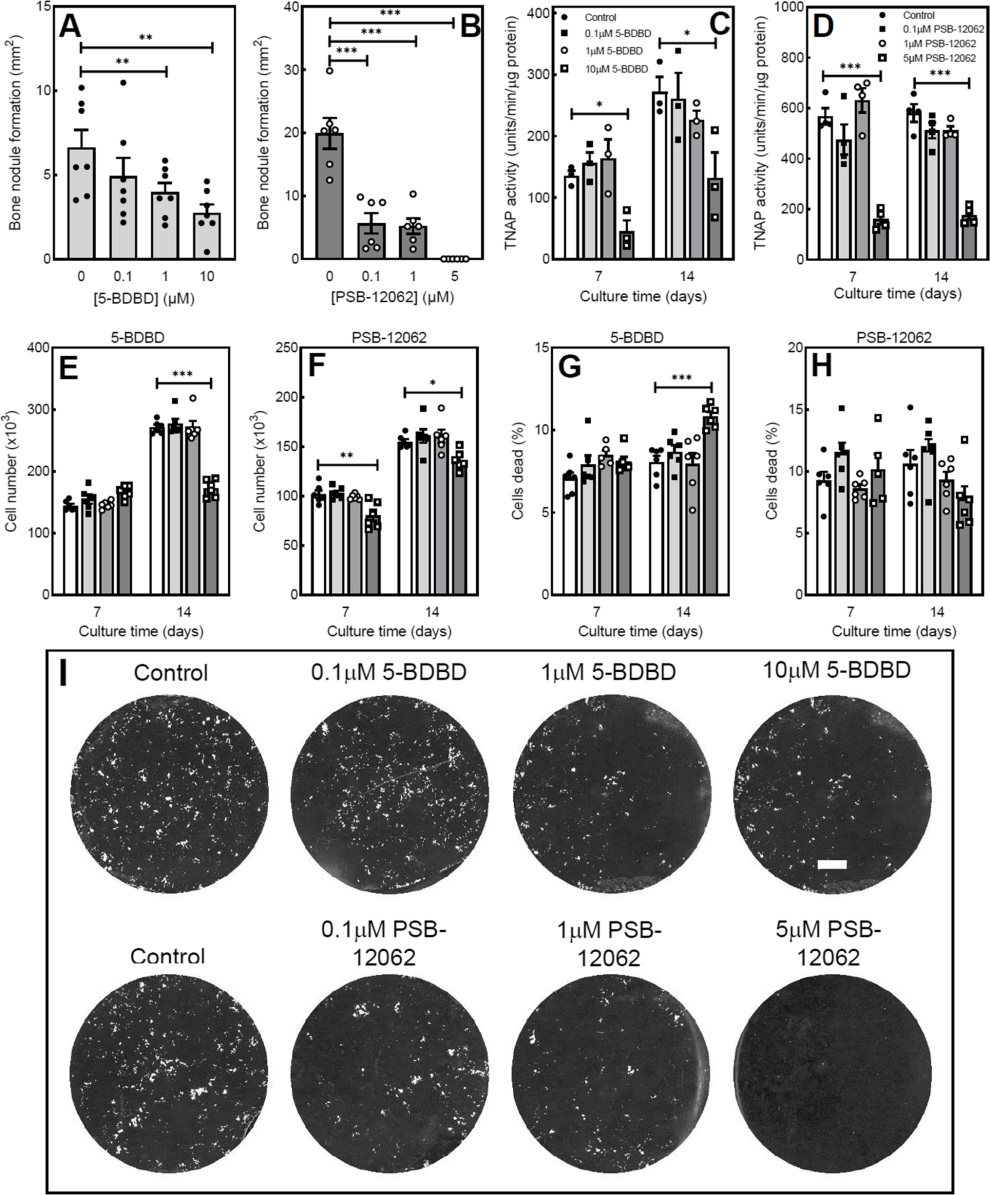
The effect of P2X4 receptor antagonists on bone formation, TNAP activity and cell number. Culture with (**A**) 5-BDBD (≤ 10 μM) and (**B**) PSB-12062 (≤ 5 μM) dose-dependently inhibited bone formation by up to 60% and 100%, respectively. (**C**, **D**) TNAP activity was reduced by up to 70% in osteoblasts treated with P2X4 receptor antagonists. Prolonged exposure to the highest dose of (**E**) 5-BDBD and (**F**) PSB-12062 decreased osteoblast numbers by 40% and 22%, respectively. (**G**, **H**) 5-BDBD but not PSB-12062 caused a small increase in the proportion of dead cells. Data shown as mean ± SEM (*n* = 3–6 independent experiments), * = *p* < 0.05, ** = *p* < 0.01, *** = *p* < 0.001. (**I**) Representative whole well scans (unstained cell layers) showing the reduced bone formation in osteoblasts cultured with 5-BDBD and PSB-12062. Scale bar = 0.5 cm

Osteoblasts cultured with 5-BDBD showed a dose-dependent (≥ 1 μM) decrease in the level of mineralised bone nodule formation (Fig. 2A and I). At the highest dose (10 μ M), bone formation was decreased by 60%. A second P2X4 receptor antagonist, PSB-12062 (0.1–1 μM), inhibited bone formation by up to 75%, with abolition at 5 μM (Fig. 2B). TNAP activity was reduced up to 70% and 50% in differentiating and mature, mineralising osteoblasts, respectively, by 10 μM 5-BDBD (Fig. 2C). PSB-12062 (5 μM) decreased TNAP activity by ~ 70% (Fig. 2D). Representative whole well scans showing the reduced bone formation in osteoblasts treated with 5-BDBD and PSB-12062 (unstained) are shown in Fig. 2I.

### Prolonged exposure to higher doses of P2X4 antagonists decreases osteoblast number

Culture with low concentrations of 5-BDBD (≤ 1 μM) had no effect on osteoblast number and viability at any stage. In mature, mineralising cells, the highest concentration of 5-BDBD (10 μM) decreased the osteoblast number by 40% and increased the proportion of dead cells by 30%. (Fig. 2E and G). PSB-12062 (5 μM) caused small decreases in osteoblast number (≤ 22%) but had no effect on cell viability (Fig. 2F and H).

### Increased oil red O staining in cells treated with 5-BDBD and PSB-12062

**Fig. 3:**
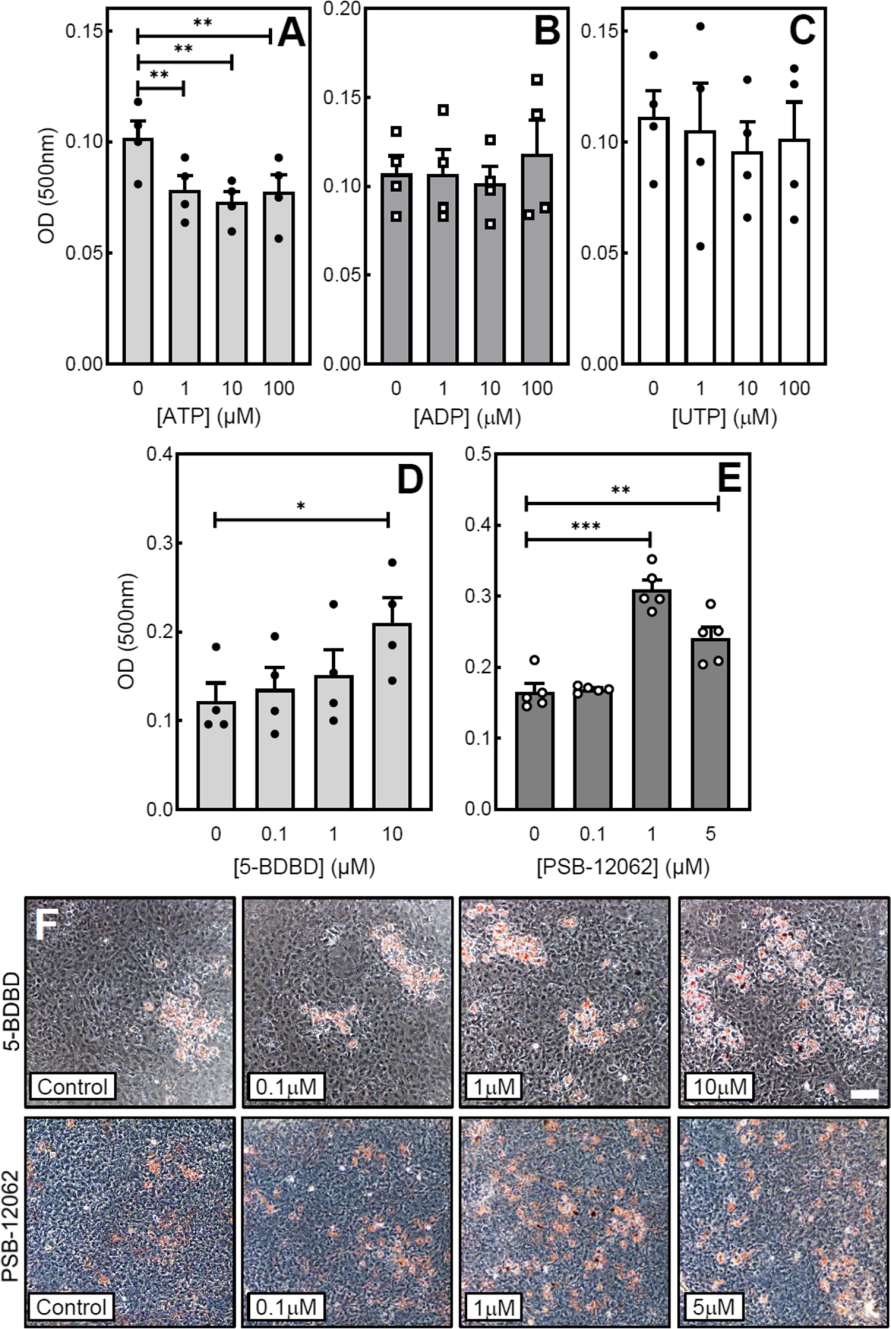
The effect of extracellular nucleotides, 5-BDBD and PSB-12062 on adipocyte formation. Culture with (**A**) ATP (≥ 1 μM) decreased adipocyte formation by up to 25%. (**B**) ADP and (**C**) UTP had no effect on the level of adipocytes. (**D**) 5-BDBD (10 μSEM (*n* = 4–5 independent experiments), * = *p* < 0.05, ** = *p* < 0.01. (**E**) Representative phase contrast microscopy images showing the increased level of oil red o staining in cells treated with 5-BDBD and PSB-12062. Scale bar = 50 μm

The universal P2 receptor agonist, ATP, decreased the level of oil red O staining in mature osteoblast cultures by up to 25% (Fig. 3A). The P2Y receptor agonists, UTP and ADP, had no effect on the amount of oil red O staining (Fig. 3B and C). Long-term culture with 5-BDBD (10 μM) or PSB-12062 (≥ 1 μM) increased the oil red O staining levels in mature osteoblasts by ≤ 80% (Fig. 3D and E). Representative phase contrast microscopy images show the higher level of oil red O staining in cells cultured with 5-BDBD and PSB-12062 (Fig. 3F).

### 5-BDBD decreases the mRNA expression of osteoblast marker genes

**Fig. 4:**
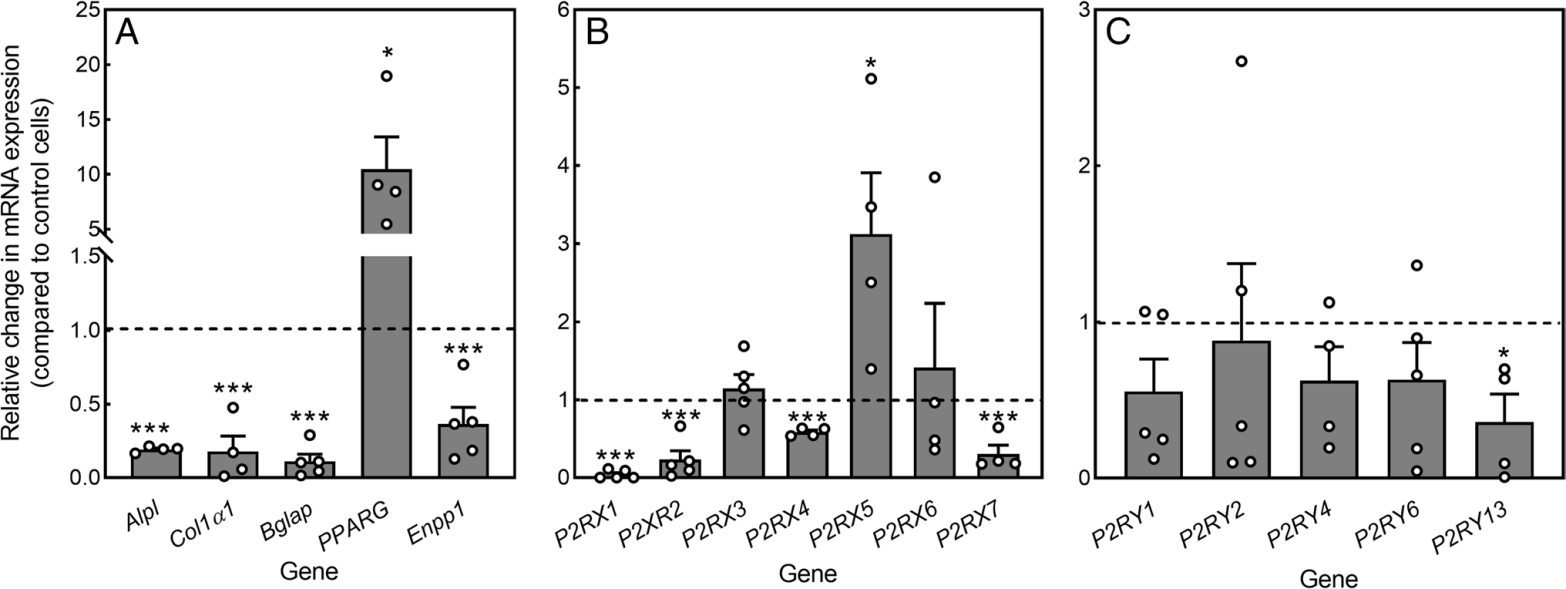
The effect of 5-BDBD on osteoblast gene expression. (**A**) In osteoblasts cultured with 5-BDBD (10 μM) expression of *Alpl, Colα1, Bglap*, and *Enpp1* was decreased, whilst levels of *PPARG* were increased tenfold. (**B**) Expression of *P2RX1, P2RX2, P2RX4*, and *P2RX7* was decreased in 5-BDBD treated cells, whereas *P2XR5* levels were threefold higher. (**C**) P2Y receptor expression was generally unaffected by 5-BDBD. Data shown as mean relative to untreated cells (represented by the dashed line) ± SEM (*n* = 4–5 RNA sets), * = *p* < 0.05, *** = *p* < 0.001

The analysis of mRNA levels in mature osteoblasts showed that culture with 5-BDBD (10 μM) decreased the expression of the marker genes *Alpl* (TNAP), *Col1α1*, *Bglap* (Ocn), and *Enpp1* (NPP1). Conversely, levels of the adipogenic transcription factor *PPARG* were increased 10-fold in 5-BDBD-treated cells (Fig. 4A). The qRT-PCR analysis also showed that 5-BDBD influenced P2X receptor mRNA expression, decreasing *P2RX1, P2RX2, P2RX4*, and *P2RX7* levels but increasing *P2XR5* expression (threefold) (Fig. 4B). P2Y receptor expression was generally unaffected by 5-BDBD, the only exception being *P2RY13*, levels of which were decreased (Fig. 4C).

### 5-BDBD does not prevent the inhibitory effects of ATP and α,β-meATP on bone mineralisation

**Fig. 5:**
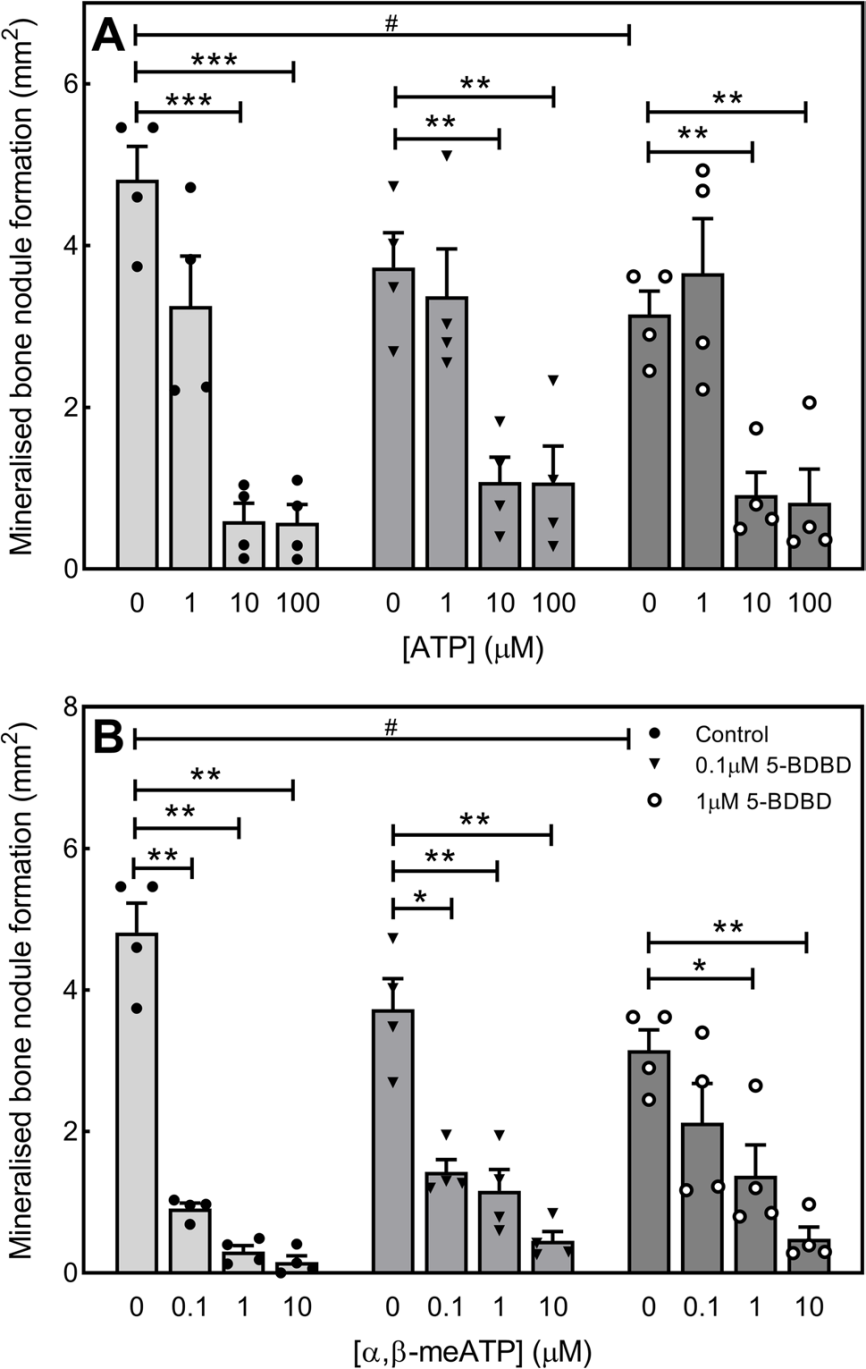
5-BDBD does not prevent the effects of ATP and α,β-meATP on bone mineralisation. The inhibitory effects of (**A**) ATP and (**B**) α,β-meATP on bone mineralisation are not prevented by 5-BDBD (≤ 1 μM). Data shown as mean ± SEM (*n* = 4 independent experiments), * = *p* < 0.05, ** = *p* < 0.01, *** = *p* < 0.001, # = *p* < 0.05

Previous work has shown that ATP and α,β-meATP acting via multiple P2 receptor subtypes inhibit bone mineralisation [15, 38]. Since these compounds are both agonists at the P2X4 receptor, this study investigated whether lower dose 5-BDBD could attenuate the actions of these nucleotides on bone mineralisation. Consistent with Fig. 2A, 1μM 5-BDBD caused a 35% decrease in bone formation compared to untreated cells. However, at this level or one that has no effect on osteoblast function (0.1 μM), 5-BDBD did not prevent the inhibitory actions of ATP and α,β-meATP (Fig. 5A and B). However, in 5-BDBD treated cells, the potency of ATP and α,β-meATP appeared reduced. For example, in control cells 100 μM ATP decreased bone mineralisation by 89% but only by 72% in the presence of 5-BDBD. Conversely, 0.1 μM α,β-meATP reduced bone mineralisation by 82% in untreated cells but in osteoblasts treated with 0.1 μM or 1 μM 5-BDBD the inhibitory effect was 62% and 35%, respectively.

### High doses of P2X4 receptor antagonists inhibit osteoclast formation and resorptive activity

**Fig. 6:**
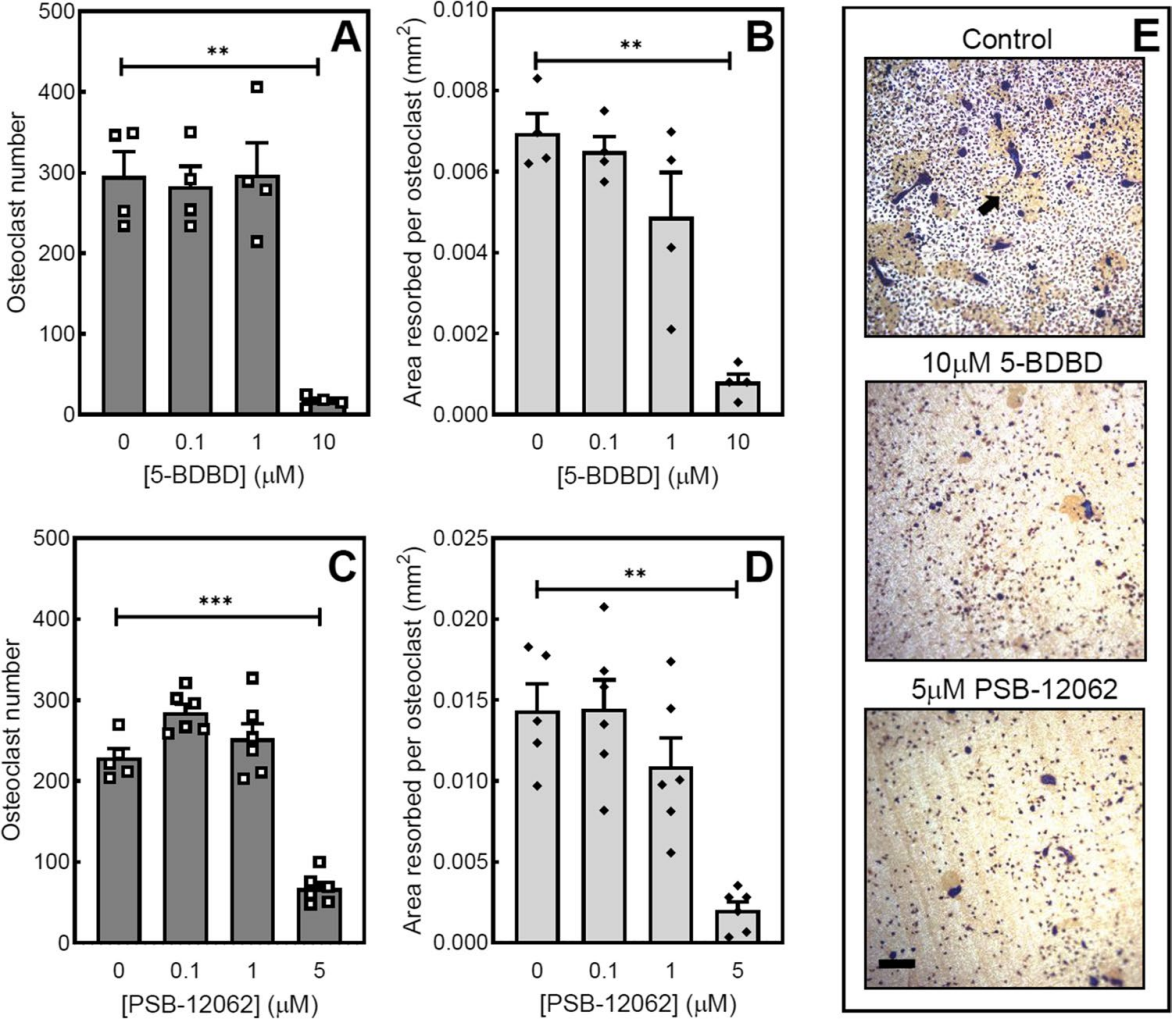
The effect of P2X4 receptor antagonists on osteoclast formation and activity. Prolonged exposure to 5-BDBD (10 μM) decreased (**A**) osteoclast formation by 95% and (**B**) resorptive activity by 90%. PSB-12062 (5 μM) reduced (**C**) osteoclast number and (**D**) bone resorption by 70% and 85%, respectively. Data shown as mean ± SEM (*n* = 4–6 independent experiments), ** = *p* < 0.01, *** = *p* < 0.001. (**E**) Reflective light microscopy images showing osteoclasts treated with 5-BDBD and PSB-12062; resorption pits are the tan areas highlighted by the arrow. Scale bar = 50 μm

Low concentrations of 5-BDBD (≤ 1 μM) had no effect on osteoclasts, whereas 10 μM 5-BDBD decreased osteoclast formation and resorptive activity by 95% and 90%, respectively. (Fig. 6A and B). PSB-12062 (5 μM) was also inhibitory reducing osteoclast number and bone resorption by 70% and 85%, respectively; no effects were seen at lower concentrations (≤ 1 μM) (Fig. 6C and D). Reflective light microscopy images illustrating the inhibitory actions of 5-BDBD and PSB-12062 on osteoclasts are shown in Fig. 6E.

## Discussion

Until recently, the lack of selective agonists and antagonists for the P2X4 receptor has hindered the study of the functional effects of this receptor in bone. This investigation used the inhibitors, 5-BDBD and PSB-12062, to establish the role of the P2X4 receptor on osteoblasts and osteoclasts. Treatment with both antagonists decreased mineralised bone nodule formation and TNAP expression and activity. 5-BDBD also reduced osteogenic gene expression whilst increasing the number of adipocytes present in the culture. High levels of both P2X4 receptor antagonists inhibited osteoclast formation and activity. Together, these data suggest for the first time that the P2X4 receptor could play a role in modulating bone cell function, particularly osteoblast differentiation.

Consistent with previous reports, this study demonstrated abundant expression of P2X4 receptor mRNA and protein by osteoblasts and osteoclasts [12, 15, 20–23]. The culture of osteoblasts with 5-BDBD (10 μM) and the more potent PSB-12062 (≥ 0.1 μM) decreased mineralised nodule formation in a concentration-dependent manner. Higher levels of these antagonists also reduced TNAP expression and activity. mRNA expression of several osteoblast-associated genes (*Alpl*, *Col1α1*, *Bglap, Enpp1*) was suppressed in cells treated with 5-BDBD. Concurrently, this P2X4 receptor antagonist increased the number of adipocytes (as shown by higher oil red O staining) and the expression of the adipogenic transcription factor, PPARγ. Furthermore, ATP (but not ADP or UTP) reduced the level of oil red O staining in osteoblast cultures. Together, these data suggest that the observed reduction in bone formation caused by P2X4 receptor inhibition is likely a consequence of reduced osteoblast differentiation. This suggests that the P2X4 receptor primarily exerts a pro-osteogenic effect on osteoblasts. In agreement, genetic analysis has shown that a loss of function polymorphism in the P2X4 receptor is associated with reduced BMD and increased risk of osteoporosis [26]. However, prolonged exposure to the highest doses of both 5-BDBD and PSB-12062 also decreased osteoblast number. Thus, cytotoxic or antiproliferative effects of these compounds could also contribute to the observed effects. It is also worth noting that the concentration range at which 5-BDBD and PSB-12062 exert their functional effects on osteoblasts is similar to that of selective antagonists acting at other P2 receptors [39, 40].

The ability of extracellular nucleotides and P2 receptors to modulate the differentiation of mesenchymal stromal cells (MSCs) into osteoblasts or adipocytes has been an area of significant study in recent years [39, 41–45]. To date, three P2X receptors (P2X5, P2X6, P2X7) and seven P2Y receptors (P2Y_1_, P2Y_2_, P2Y_4_, P2Y_6_, P2Y_11_, P2Y_13_, P2Y_14_) have been associated with this process [39, 41–45]. A range of experimental models utilising cells from different species (e.g. human, rodent), tissues (e.g. adipose, bone marrow, calvaria), and differentiation states (e.g. MSC, osteoprogenitor) are used to study the transition from MSC-to-osteoblast/adipocyte [39, 41–45]. This makes a direct comparison between studies challenging and could potentially explain the high number of P2 receptors associated with this process. It also illustrates that the role of purinergic signalling in MSC differentiation is complex and likely to be age, species, and context dependent. This work used cells derived from neonatal rodent calvaria, an approach, which isolates a heterogeneous cell population comprising mainly of osteoprogenitors already committed to the osteogenic lineage [31]. Treatment with P2X4 receptor antagonists slowed osteoblast differentiation, and this was reflected by the reduced bone formation and osteogenic gene expression. They also caused a modest increase in adipocyte formation. Thus, it is possible that the effect of P2X4 inhibition could be more pronounced when applied to cells that are at an earlier stage of lineage commitment. Therefore, additional work using MSCs would be beneficial to fully understand the role of the P2X4 receptor in modulating osteogenic differentiation.

Previous work has shown that the P2X4 receptor main agonists, ATP and α,β-meATP, potently inhibit bone mineralisation [10, 15, 38]. Whilst ATP can activate most P2 receptors to some extent, α,β-meATP is only an agonist at P2X receptors. Pharmacological analysis suggested that α,β-meATP is most likely acting via the P2X1 and/or P2X7 receptors [15]. In this study, low-dose 5-BDBD did not prevent the inhibitory effects of ATP and α,β-meATP. However, the potency of both compounds was reduced in the presence of 5-BDBD. Thus, the inhibitory effects of ATP and α,β-meATP are, in keeping with earlier studies, most likely mediated by the P2X1 and/or P2X7 receptors [15]; however a very minor role of the P2X4 receptor cannot be excluded.

Prolonged culture with 5-BDBD (10 μM) and PSB-12062 (5 μM) had a profound inhibitory effect on both osteoclast number and resorptive activity. This suggests that this receptor, like other P2 receptors [7, 11, 39], may act to modulate osteoclast formation and function under normal conditions. Interestingly, the P2X4 receptor is thought to be predominantly localised on lysosomes, where it may modulate their function [46]. Lysosomes play an important role in bone resorption, therefore the decreased resorption observed here could, in part, be a consequence of reduced lysosomal function. Nonetheless, these data should be interpreted with caution as neither antagonist acted in a dose-dependent manner and the reduction in osteoclast formation was associated with a decrease in precursor cells. Consequently, it is possible that the inhibitory effects observed reflect general cytotoxic effects of these compounds rather than a P2X4 receptor-specific effect.

A recent study provided the first overview of the skeletal phenotype of the P2X4 receptor knockout mouse [29]. Ellegaard et al. reported that mice lacking the P2X4 receptor displayed increased trabecular and cortical bone mineral density (BMD) in an age-dependent manner. The in vitro data presented here suggest receptor removal would lead to less bone formation and resorption. The increased bone mass in the P2X4 knockouts indicates that the defect in bone resorption is greater than in bone formation shifting the remodelling balance in favour of net bone gain. However, the skeletal phenotype results are potentially confounded by the observation that the wildtype but not the knockout animals possess a P451L passenger mutation in the P2X7 receptor gene. Whether this mutation has any impact on the observed skeletal phenotype of the P2X4 knockout mice is unclear. However, it has been shown that the ability of ATP to induce pore formation differs depending on whether the mutant (451L) or wildtype (451P) allele is present [47]. Thus, additional studies are required to fully understand the role of the P2X4, and its interactions with the P2X7 receptor, on bone mass in vivo.

The findings presented in this study provide the first indication of how the P2X4 receptor modulates bone cell function. However, several factors potentially confound our understanding of how this receptor regulates bone remodelling. For example, the P2X4 receptor can exist in a homomeric or heteromeric form (e.g. P2X4/6). These homo- and heteromultimers are likely to have different pharmacology and, potentially, downstream functional effects. Furthermore, the gene encoding the P2X4 receptor is located in close proximity to the gene encoding the P2X7 receptor on chromosome 12, and they are thought to share the same ancestral gene [19]. Taken together, it is clear that this receptor has a potentially important, but as yet not fully defined, role in bone.

## Data Availability

Data is available on request from the authors.
